# Promising Support Coming from Nature: Antioxidant and Anti-Inflammatory Potential of *Castanea sativa* Wood Distillate on Skin Cells

**DOI:** 10.3390/cimb46090556

**Published:** 2024-08-26

**Authors:** Arianna Filippelli, Valerio Ciccone, Stefano Loppi, Lucia Morbidelli

**Affiliations:** 1 Laboratory of Pharmacology of Angiogenesis and Microcirculation, Department Life Sciences, University of Siena, Via A. Moro 2, 53100 Siena, Italy; arianna.filippelli@student.unisi.it (A.F.); valerio.ciccone@unisi.it (V.C.); 2 BioAgry Laboratory, Department Life Sciences, University of Siena, Via P.A. Mattioli 4, 53100 Siena, Italy; stefano.loppi@unisi.it; 3 Interuniversity Center for Studies on Bioinspired Agro-Environmental Technology (BAT Center), 80055 Naples, Italy

**Keywords:** inflammation, oxidative stress, skin protection, wound healing, endothelium, natural product

## Abstract

Tissue homeostasis, function recovery, and protection mechanisms are boosted by the balanced and timely control of inflammation and oxidative stress. Nowadays, many natural products and bio-derivates exhibit antioxidant and anti-inflammatory activity, supporting medical care and tissue wellness against inflammation, oxidative stress, and inflammaging. *Castanea sativa* wood distillate (WD) is a bio-derivative used as a corroborant and biofertilizer in agriculture. Based on the safety profile of low concentrations of WD on human cells, the present study aims to assess the anti-inflammatory and antioxidant activity of WD on different cell types in the integumentary system. Human keratinocytes, mucosal epithelium, dermal fibroblasts, and endothelial cells were exposed to WD, and the concentrations devoid of pro-apoptotic potential were profiled. Then, the effect of nontoxic doses of WD revealed an anti-inflammatory effect, observed through the immunodetection of prostanoid cascade markers in experimentally induced inflammation. A reduction in endothelial hyperpermeability was evidenced by the immunofluorescence analysis of cell–cell adhesion proteins, VE-cadherin and ZO-1. In addition, WD buffered the exogenously produced oxidative stress. On the whole, WD showed both anti-inflammatory and antioxidant activities on the various cell types, preserving endothelial barrier integrity. Overall, this study supports the involvement of this bio-derivative in novel exploitable fields, such as therapeutic dermatological applications for human and animal medical care.

## 1. Introduction

Inflammation and oxidative stress are key mechanisms in tissue homeostasis. However, elevated and persistent levels of reactive oxygen species (ROS) and inflammatory cytokines can lead to pathological conditions [1,2]. These processes regulate cutaneous functions, such as tissue renewal, regeneration, and wound healing, which are complex processes orchestrated by inflammation and chemokines [3,4]. Conversely, the interplay between increased oxidative stress and inflammation converges during the inflammaging process, causing accelerated senescence and the aging of tissues, such as skin and the endothelium [5,6,7,8]. The richness in polyphenols and tannins of various natural extracts or preparations has been extensively reported to reduce inflammation, oxidative stress, and promote tissue repair, justifying their use in food and medicinal applications [9]. Plant-derived tannins retain more hydroxyl groups, making them more prone to oxidation and, thus, exhibiting higher antioxidant activity [10]. Likewise, polyphenols show antioxidant capacity by suppressing ROS formation. They act through the inhibition of the enzymes involved in ROS production, direct scavenging, and the upregulation of antioxidant defenses [11]. Additionally, tannins extracted from different plants display anti-inflammatory activity, by interfering with the overproduction of nitric oxide (NO) and prostaglandin-E2 (PGE2) [12]. Polyphenols share these anti-inflammatory properties, activating the protective transcription factor, Nrf2, and interfering with enzymatic systems and signaling pathways involved in T cell proliferation and B lymphocyte activation. Furthermore, polyphenols modulate the key enzymes involved in arachidonic acid processing and prostanoid synthesis, such as cyclooxygenase-2 (COX-2) and microsomal prostaglandin E synthase-1 (mPGES-1) [1,13]. The potential antioxidant and anti-inflammatory action of plant products may be utilized to treat or prevent pathological processes, such as acute or chronic inflammation, bacterial infection-induced inflammation, and oxidative stress, particularly affecting the cardiovascular and cutaneous systems [14,15].

In this scenario, we are investigating pyroligneous acid, also known as wood distillate or wood vinegar (WD), extracted from residual virgin sweet chestnut (*Castanea sativa*) biomass [16,17]. In recent years, WD has been broadly used for its beneficial effects in agriculture, and its indirect nutraceutical properties have recently been recognized [18]. Notwithstanding its promising composition and the broad spectrum of beneficial proprieties [18,19], the complete profiling of WD is still ongoing using a cross-disciplinary research approach [17,18,19,20,21]. Previously, we deeply investigated and defined the safety profile of this bio-derivative and the possible implications for human cells [17]. Given its stable composition rich in phenols, polyphenols, and tannins, the potential antioxidant and anti-inflammatory action of WD may represent a novel application of this bio-derivative. Therefore, this study aims to investigate the anti-inflammatory and antioxidant capabilities of non-cytotoxic concentrations of WD, in an in vitro model of skin and mucosa tissues, as well as on the vascular endothelium, to mimic transcutaneous absorption, blood distribution, and skin and mucosa care. This research highlights the importance and the concrete potential of integrating natural products with modern medical research.

## 2. Materials and Methods

### 2.1. Chemicals and Reagents

Wood distillate, BioDea^®^, was supplied by Bio-Esperia Srl (Umbertide, Perugia, Italy), extracted by controlled pyrolysis up to 70 °C, exploiting physiological lymphatic water, and following a temperature gradient. The preliminary extract is left to decant for almost three months to obtain an aqueous liquid fraction rich in phenols, polyphenols, and tannins (10–13%) [16,17]. The comprehensive characterization of the tested WD, encompassing a detailed profile of all its components, was first reported by Celletti et al. [20]. This work was subsequently summarized by Maresca and coworkers [18], highlighting WD’s richness in phenolic compounds, among other important constituents. The company guarantees the stability and standardization of WD’s composition over time. Table 1 presents the detailed physio-chemical characterization of wood distillate, as published by Celletti [20] and tested in our research.

The human recombinant Interleukin 1 beta (IL-1β), Tumor Necrosis Factor-α (TNF-α), and H_2_O_2_ were purchased from Sigma-Aldrich (St. Louis, MO, USA).

The antibodies against COX-2 and mPGES-1 were from Cayman Chemical (Arcore, Milan, Italy); Caspase 3, cleaved Caspase 3, vascular endothelial cadherin (VE-cadherin), and zonula occludens-1 (ZO-1) were purchased from Cell Signaling technologies (Danvers, MA, USA); intercellular adhesion molecule 1 (ICAM-1-1) and vascular cell adhesion molecule 1 (VCAM-1-1) were purchased from Thermo Fisher Scientific (Milan, Italy); and β-actin was purchased from Sigma-Aldrich (St. Louis, MO, USA). The secondary antibodies goat anti-rabbit IgG and anti-mouse were purchased from Merck KGaA (Darmstadt, Germany), while Alexa Fluor 488 and 555 secondary antibodies were purchased from Thermo Fisher Scientific (Waltham, MA, USA).

### 2.2. Cell Cultures

Human immortalized keratinocytes, HaCaT cells, were obtained from Thermo Fisher Scientific (Waltham, MA, USA). To mimic mucosa, human epidermoid carcinoma A431 cells (ATCC, American Type Culture Collection, Manassas, VA, USA) were used. Normal human dermal fibroblasts and human umbilical vein endothelial cells, named, respectively, NHDF and HUVEC, were supplied by Lonza (Basel, Switzerland).

HaCaT and A431 cells were grown in Dulbecco’s modified Eagle’s medium (DMEM 4500 mg/L, Euroclone, Milan, Italy), supplemented with 10% fetal bovine serum (FBS, Euroclone, Milan, Italy). NHDF were cultured in fibroblast growth medium 2 (FGM-2 Lonza, Basel, Switzerland), supplemented with 10% FBS (Hyclone, Celbio, Milan, Italy), and HUVEC were grown in endothelial growth medium (EGM-2, Lonza, Basel, Switzerland), with 10% FBS (Hyclone, Celbio, Milan, Italy), on gelatin-coated plastic. Each medium was completed with 2 mM glutamine, 100 units/mL penicillin, and 0.1 mg/mL streptomycin (Sigma-Aldrich, St. Louis, MO, USA). Cells were cultured in Petri dishes, 10 cm in diameter, up to a confluent state, in a humidified incubator with 5% CO_2_. Cells were expanded by splitting 1:6 twice a week for HaCaT and A431, while it was 1:3 twice a week for NHDF and HUVEC. HaCaT and A431 were used until passage 30, NHDF until passage 7, and HUVEC until passage 5.

### 2.3. Western Blot for Cytotoxicity Analysis

To assess the expression of the proteins involved in apoptosis, Western blot analysis for Caspase 3 cleavage was performed. Sub-confluent cells were starved for 5 h. Cells were then exposed to fresh media, with 1% FBS added. To evaluate the activation of Caspase 3, the final event of apoptosis, the cells were treated with increasing concentrations of WD, ranging between 0.04 and 0.33% (*v*/*v*) for 4 h, a time coherent with the biochemical processing of Caspase 3. Protein extraction and Western blot analysis were performed, as previously described [22]. Moreover, 50 μg of proteins for each sample were subjected to electrophoresis in 4–12% Bis-Tris gels (Life Technologies, Carlsbad, CA, USA). Proteins were blotted onto nitrocellulose membranes and then incubated overnight with primary antibodies: anti-Caspase 3 (9662S, Cell Signaling Technology, Danvers, MA, USA), anti-cleaved Caspase 3 (9664S, Cell Signaling Technology, Danvers, MA, USA), and anti-β-actin (A5441, Sigma-Aldrich St. Louis, MO, USA). The detection was carried out using the enhanced chemiluminescence system (Bio-Rad, Hercules, CA, USA). For each sample, the ratio of arbitrary densitometry units (A.D.Us.) of cleaved Caspase 3/A.D.Us. of total Caspase 3 was calculated. Data were normalized using β-actin and presented as mean ± SD of at least three experiments.

### 2.4. Alkaline Comet Assay

The genotoxic effect of WD was excluded using the alkaline comet, which assesses single-strand damage of cellular DNA [23]. A suspension of 1 × 10^6^ cells/mL was treated for 6 h with pure and diluted WD, used at concentrations ranging between 0.04 and 0.5%, (*v*/*v*). The effect of WD was compared to H_2_O_2_ [50 µM], taken as a positive control of genotoxicity. At the end of the stimulation, the cells were mixed with low-melting-point agarose (LM Agarose, SeaPlaque^®^, Lonza, Rockland, ME, USA), and 80 µL of the solution was dispensed onto a glass slide, precoated with normal-melting-point agarose (NM Agarose, SeaKem^®^ Lonza, Rockland, ME, USA). The samples, embedded into an agarose sandwich, were exposed to a basic lysis solution (pH 10) for 1 h in the dark at room temperature (RT). A horizontal electrophoresis run was then performed up to 60–100 V, at 300 mA, in an alkaline buffer, for 30 min at 4 °C. Glasses were washed twice with Tris HCl (Sigma-Aldrich, St. Louis, MO, USA), a neutralization buffer, and the DNA was fixed with pure EtOH. The samples were exposed to genomic dye DAPI [10 µg/µL] for 3 h at 4 °C, in the dark. Comet-like structures, formed by the damaged DNA, were analyzed by a Nikon Eclipse TE 300 microscope (Nikon, Tokyo, Japan), equipped with a digital camera and NIS Element software A R 3.0, at 40× magnification.

### 2.5. Permeability Assay

Endothelial cells were seeded on gelatin-coated transwell inserts (0.4 µm pores, Corning, Lowell, MA, USA), at a density of 8 × 10^4^ cells/insert [24]. The inserts were placed onto 12 multiwell plates and incubated at 37 °C and in 5% CO_2_. Confluent monolayers of HUVEC were treated with non-cytotoxic concentrations of WD [0.04–0.07%, (*v*/*v*)] for 18 h and an inflammatory mix (MIX: IL-1β [100 ng/mL] + TNF-α [10 ng/mL]), alone or in combination with WD. FITC–dextran [10 µM], used as a fluorescent marker of permeability, was added on top of the cell monolayers. Permeability in the HUVEC monolayer was detected as passage of FITC–dextran from the upper to the lower compartments, which was measured at different times, namely 15, 45, and 60 min. The extent of the permeability was determined by measuring the fluorescence at 485/535 nm (excitation/emission), using a microplate reader (Infinite 200 Pro, Spectra Fluor, Tecan Life Sciences, Männedorf, Switzerland). The data about the paracellular flux were reported as fluorescence units (mean ± SD, n = 3).

### 2.6. Immunofluorescence of Inflammatory and Adhesion Markers on HUVEC

A suspension of 5 × 10^4^ cells/mL was seeded onto 1 cm circular glass coverslips, precoated with gelatin. After adhesion, the cells were treated with non-cytotoxic concentrations of WD [0.04–0.07%, (*v*/*v*)] in the presence or absence of the inflammatory mix (MIX: IL-1β [100 ng/mL] + TNF-α [10 ng/mL]). The treatment was prolonged for 18 h, then an indirect immunofluorescence analysis was performed. Cells were fixed in 4% paraformaldehyde (PFA; Thermo Fisher Scientific, Milan, Italy) for 10 min or cold acetone (Sigma-Aldrich, St. Louis, MO, USA) for 5 min, washed in PBS and incubated with goat serum (Euroclone, Pero, Milan Italy) for 1 h at RT. Fixed cells were then incubated overnight at 4 °C with primary antibodies, diluted 1:50 in PBS, containing 0.5% goat serum [24]. To evaluate the anti-inflammatory action of WD, fixed cells were exposed to anti-COX-2 and anti-mPGES-1 antibodies (Cayman Chemical, Arcore, Milan, Italy). Antibodies versus ZO-1 and VE-cadherin (Cell Signaling Technologies, Danvers, MA, USA), and ICAM-1 and VCAM-1 (Thermo Fisher Scientific, Milan, Italy) were used to investigate, respectively, the modulation of endothelial junctions and leukocyte adhesion proteins in endothelial cells. Then, incubation for 1 h was performed with the secondary antibodies: Alexa Fluor 488, goat anti-rabbit (for ZO-1); Alexa Fluor 488, goat anti-mouse (for COX-2 and VCAM-1); Alexa Fluor 555, goat anti-rabbit (for mPGES-1 and VE-cadherin); and Alexa Fluor, goat anti-mouse (for ICAM-1). At the end, the cells were washed, and the coverslips mounted with mounting medium (Sigma-Aldrich, St. Louis, MO, USA). Images of the cells were taken using a Nikon Eclipse TE 300 microscope (Nikon, Tokyo, Japan) with a digital camera and NIS Element software A R 3.0, at 10–60× magnification. The immunofluorescence intensity was quantified by FIJI ImageJ software 6.1.1.jar and reported as arbitrary densitometry units (A.D.Us.)/counted nuclei. The data are presented as mean ± SD of at least three experiments.

### 2.7. ROS Measurements

In order to assess the oxidative stress, the ROS levels were measured. A suspension of 1.5 × 10^3^ cells/100 µL, for each cell type, was seeded in 96-multiwell plates and, after adherence, the cells were quickly (30 min, 1 h, and 4 h) treated with increasing concentrations of WD [0.02–0.14% (*v*/*v*)], alone or in combination with H_2_O_2_ [50 µM], which is considered a positive inducer of oxidative stress. The fluorophore 2,-7-dichlorodihydrofluorescein diacetate (DCFH2-DA) (Invitrogen, Milan, Italy) was added in a medium without phenol red (10 μM, 30 min). The intracellular levels of ROS were evaluated with a microplate reader (excitation/emission 495/527; Infinite 200 Pro, Spectra Fluor, Tecan Life Sciences, Männedorf, Switzerland). The results are reported as the relative fluorescence units (RFUs) corrected for the number of cells, once fixed, stained, and counted.

### 2.8. Scratch Assay

To evaluate the migration of adherent cells treated with WD, a scratch assay was performed. HaCaT and NHDF (2 × 10^5^ cells/mL) were seeded in 24 multiwell plates, in a medium with 10% FBS, up to a confluent state. A scratch was mechanically performed on the layer of the cells, then treated with increasing concentrations of WD [0.02–0.14% (*v*/*v*)], diluted in a medium with 1% or 5% FBS added. Untreated cells in a medium with 0.1% and 10% FBS were analyzed, respectively, as negative and positive controls of migration. The antimitotic compound cytosine arabinoside (ARA-C) [2.5 μg/mL] was added to all the wells. Images of the wound in each well were acquired from 0 to 18 hours using a phase contrast microscope (Nikon Eclipse TE 300, Nikon, Tokyo, Japan), at 10× magnification. The rate of migration was calculated by quantifying the area of the wound at the starting time and after 18 h. The results are expressed as the percentage of the area of the wound relative to the time control (untreated cells or 0% WD).

### 2.9. Statistical Analysis 

The experimental data are presented as the mean ± SD of at least three independent experiments. Data normality was checked with the Shapiro–Wilk test and the equality of variance with the Levene test. A one-way ANOVA, followed by Bonferroni’s multiple comparisons test, were used to evaluate the differences among the groups. A value of *p* < 0.05 was considered statistically significant. All the statistical analyses were performed using GraphPad Prism version 8.3.0 for Windows.

## 3. Results

### 3.1. Characterization of the Safety Profile of WD on Skin Cell Types and the Endothelium

**Figure 1:**
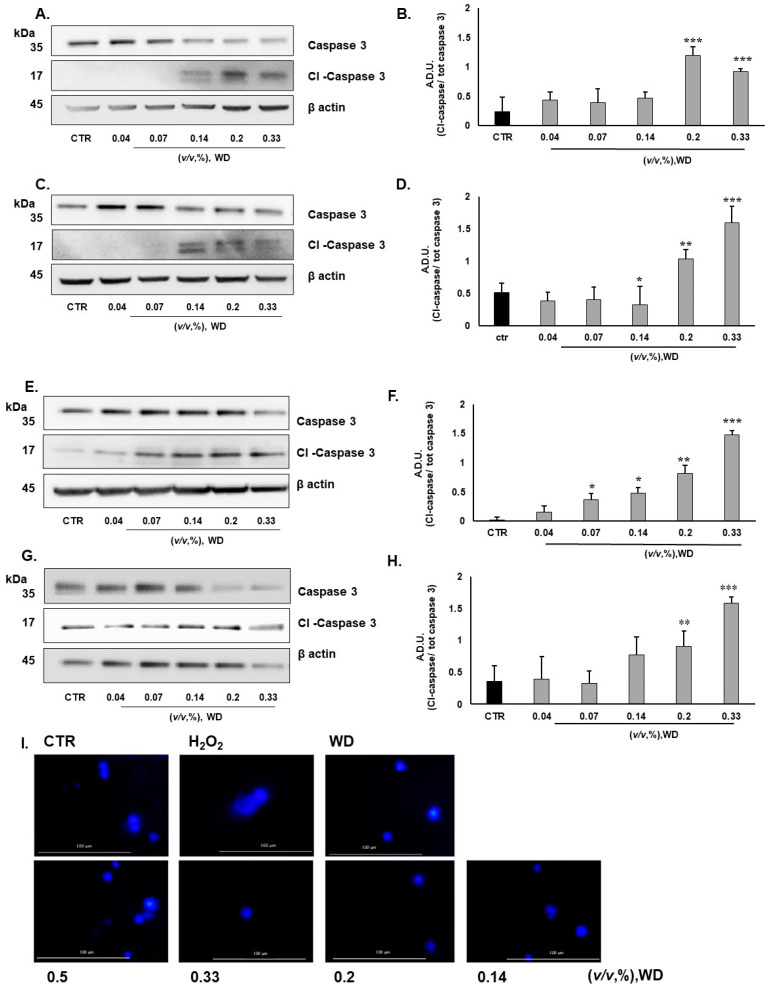
Assessment of cytotoxicity and genotoxicity of WD on skin cells: HaCaT (**A**,**B**), A431 (**C**,**D**), NHDF (**E**,**F**), and HUVEC (**G**,**H**) were exposed to increasing concentrations of WD [0.04–0.33%, (*v*/*v*)] in a medium supplemented with 1% FBS. Caspase 3 cleavage was assessed after 4 h of treatment. Signals were evaluated through Western blot analysis and β-actin was used as a reference. For each experimental condition, arbitrary densitometry units (A.D.Us.) ± SD are reported as cleaved Caspase 3/total Caspase 3, and β-actin was used as a loading control (n = 3). * *p* < 0.05, ** *p* < 0.01, and *** *p* < 0.001 vs. untreated cells (CTR). (**I**) The alkaline comet assay was performed on HUVEC to evaluate the potential genotoxicity of WD. Cells were exposed to pure or diluted WD [0.14–0.5%, (*v*/*v*)] and H_2_O_2_ [50 μM] for 2 h. Agarose electrophoresis and exposure to the alkaline buffer were performed. Nuclei were visualized by staining with DAPI, and fluorescence analysis was conducted at high magnification (60×). The presented images are representative of n = 3 experiments.

According to the previously published data about WD-affected cell viability [17], the apoptotic behavior of cells has been investigated here by the assessment of Caspase 3 activation. Keratinocytes (HaCaT), dermal fibroblasts (NHDF), a mucosal cell model (A431), and endothelial cells (HUVEC) were exposed to WD for 4 h. A dramatic increase in cleaved Caspase 3 was observed after treatment with [0.14% (*v*/*v*)] of WD, both in HaCaT (Figure 1A,B) and in A341 cells (Figure 1C,D). Conversely, NHDF (Figure 1E,F) and HUVEC (Figure 1G,H) showed less sensitivity to WD, with significant Caspase 3 activation occurring at concentrations between 0.2% and 0.33% (*v*/*v*). In all cell types, the cleavage of Caspase 3 induced by higher concentrations of WD (0.14–0.33% (*v*/*v*) corresponded to a reduction in the total Caspase 3 levels.

To exclude the potential genotoxic effect of WD, an alkaline comet assay was performed on HUVEC. As expected, a comet-like structure was observed in the nuclei of cells treated with H_2_O_2_ [50 μM]. The formation of comet tails was not observed in the nuclei of cells treated with pure or diluted WD: the integrity and shape of the nuclei were comparable to the control condition (Figure 1I). Overall, low concentrations [0.04–0.14% (*v*/*v*)] of WD can be considered safe, since they did not induce Caspase 3 activation or genotoxicity in the various skin and mucosa cell types.

### 3.2. WD Did Not Affect the Permeability of HUVEC and Decreased the Inflammation-Mediated Permeability, Retaining the Integrity of Tight Junctions

**Figure 2:**
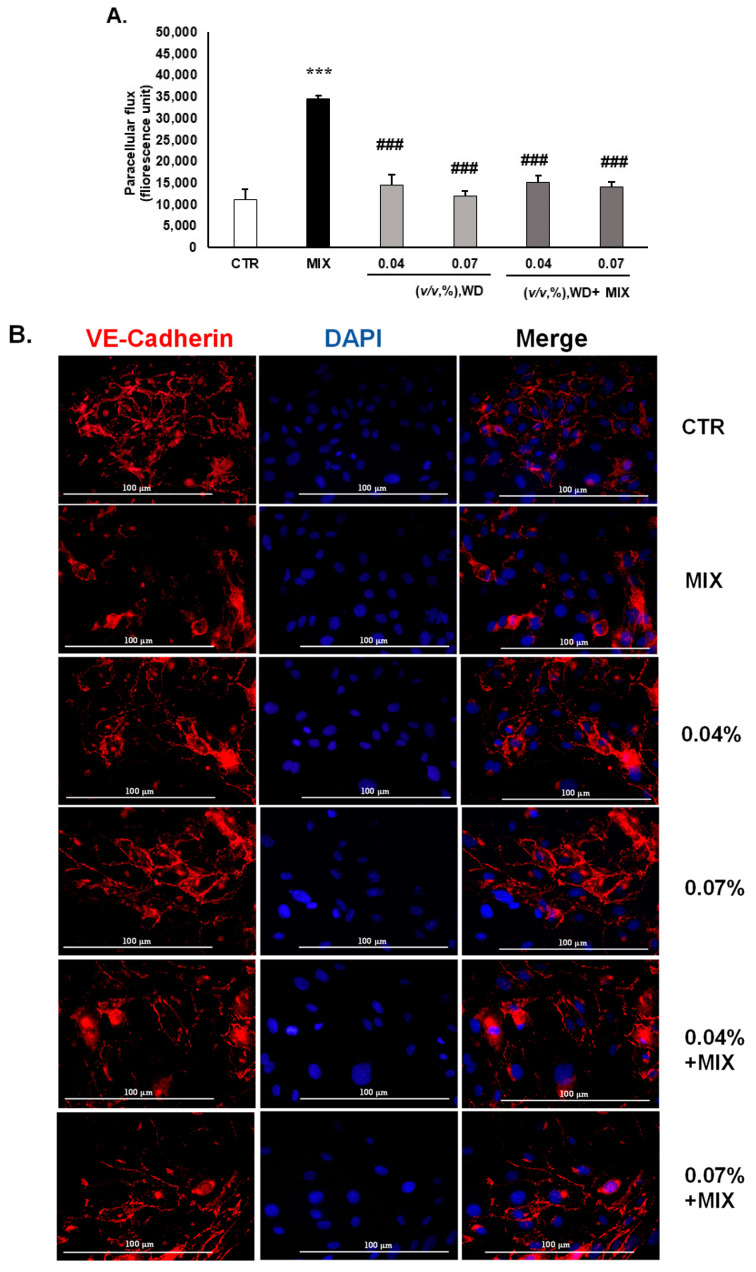

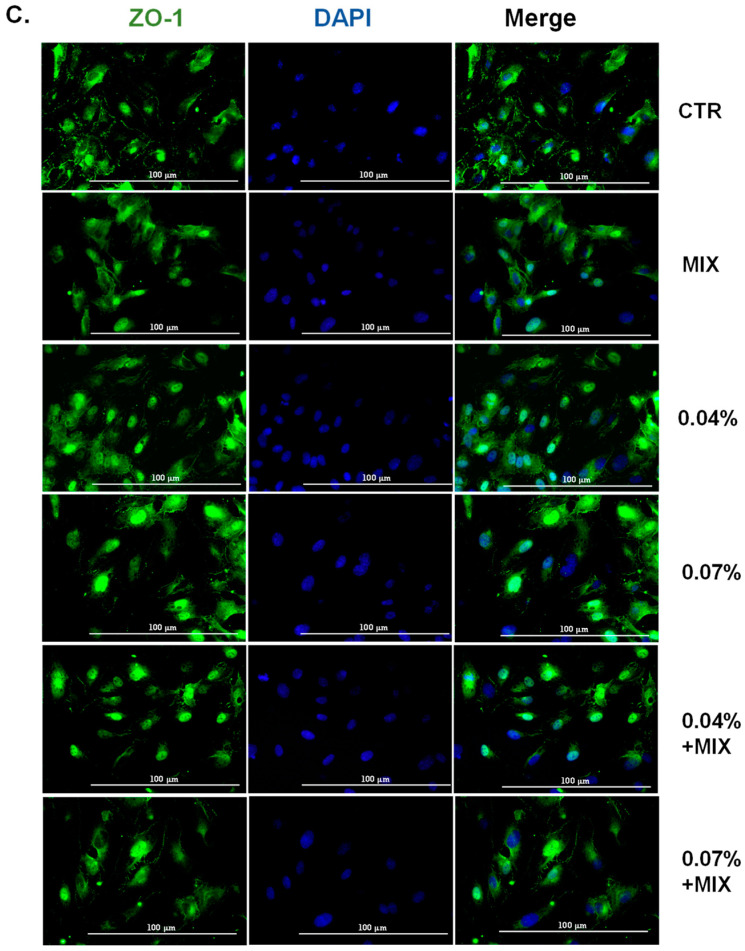
Effect of WD on endothelial paracellular permeability in HUVEC and integrity of tight junctions affected by inflammatory damage. (**A**) The permeability assay was performed on HUVEC seeded on transwell inserts. The confluent monolayers were treated with WD [0.04–0.07%, (*v*/*v*)] and an inflammatory mix (MIX: IL-1β [100 ng/mL] + TNF-α [10 ng/mL]). At the end of the treatment, FITC–dextran (10 µM) was added and permeability in the HUVEC monolayer was detected as passage of FITC–dextran from the upper to the lower compartment of the transwell insert. The extent of permeability was determined by measuring the fluorescence at 485/535 nm, excitation/emission, respectively, after 15 min. Data are reported as fluorescence unit (n = 3) ± SD; *** *p* < 0.001 vs. untreated cells (CTR); ### *p* < 0.001, compared to the MIX. From a molecular point of view VE-cadherin (**B**) and ZO-1 (**C**) were assessed by immunofluorescence analysis of HUVEC treated for 18 h with WD [0.04–0.07%, (*v*/*v*)], alone or in combination with the inflammatory mix (MIX: IL-1β [100 ng/mL] + TNF-α [10 ng/mL]. VE-cadherin fluorescence was captured by a secondary antibody conjugated with Alexa Fluor 555 (**B**). ZO-1 was visualized by Alexa Fluor 488 (**C**). DAPI-stained nuclei in blue. Images were obtained by a Nikon Eclipse TE 300 microscope (magnification 60×). The merge images were obtained using FIJI ImageJ software 6.1.1.jar. The presented pictures are representative of n = 3 experiments.

To investigate the role WD in an inflammatory environment, endothelial cells (HUVEC) were exposed to inflammatory conditions, induced by an exogenously added cytokine mix: IL-1β [100 ng/mL] + TNF-α [10 ng/mL]) (named MIX). Safe concentrations of WD [0.04–0.07%, (*v*/*v*)] were tested in this model. After 18 h of treatment of the endothelial cells with the MIX, the FITC–dextran paracellular flux increased after 15, 45, and 60 min. No significant improvement in the paracellular flux was detected after treatment with WD [0.04–0.07%, (*v*/*v*)], compared to the control conditions (Figure 2A and Figure S1A,B). Conversely, a drastic reduction in the MIX-induced hyperpermeability was observed after co-treatment with WD for all the times analyzed (Figure 2A and Figure S1A,B for 15, 45, and 60 min, respectively). With a prolonged time of exposure (60 min), the protective effect exerted by WD mildly decreased (Figure 2C), suggesting a fast cellular effect.

To evaluate the integrity of the endothelial monolayer, ZO-1 and VE-cadherin localization were analyzed [24]. The inflammatory conditions impaired the integrity of the HUVEC monolayer, as demonstrated by the fading of the fluorescence intensity of these cell–cell markers (Figure 2B,C). Coherently, ZO-1 lost its involvement in intercellular adhesion, being localized in the inner part of the cells (Figure 2B). In the basal condition, WD treatment [0.04–0.07%, (*v*/*v*)] did not affect the localization of VE-cadherin and ZO-1 (Figure 2A,B). Interestingly, the treatment of endothelial cells with WD in combination with the inflammatory MIX restored a condition comparable to the control (CTR), with VE-cadherin mainly located at the cell–cell adhesion junction (Figure 2B) and ZO-1 at the membrane and cytoplasmatic level (Figure 2C). Collectively, these data enforce the ability of WD to avoid inflammation damage on endothelial cells, revealed by hyperpermeability, and to maintain the integrity and linearity of endothelial cell–cell junctions.

### 3.3. WD Interfered with the Prostanoid Enzymatic Cascade and Adhesion Molecules in the Endothelium

Low concentrations of WD [0.04–0.07%, (*v*/*v*)] were tested on the cell lines analyzed, and the levels of the main inflammatory markers, COX-2 and mPGES-1, were assessed by immunofluorescence detection. 

An increased level of COX-2 and mPGES-1 was found in the endothelial cells (HUVEC) treated with the MIX, used as an inducing stimulus, for 18 h. A reduction in the main inflammatory markers in cells treated with inflammatory cytokines (MIX) and WD [0.04–0.07%, (*v*/*v*)] was evident, especially in cells exposed to 0.07% (*v*/*v*) of WD (Figure S2A,B and Table 2). Correspondingly, mPGES-1 expression was increased in the presence of the MIX, while WD reverted the inflammatory phenotype, reducing mPGES-1 expression at the concentration of 0.07% (Figure S2A,B and Table 2).

Next, the expression of ICAM-1 and VCAM-1 was investigated, as a consequence of inflammation occurrence. ICAM-1 and VCAM-1 labelling was not enhanced by the exposure to low concentrations of WD [0.04–0.07%, (*v*/*v*)] for 18 h (Figure S3A,B). The localization of VCAM-1 and ICAM-1 was strongly decreased by WD alone, compared to the MIX. The combined treatment with inflammatory cytokines and WD reduced the amount of both the adhesion proteins compared to cells exposed to the inflammatory MIX, with 0.07% being more effective than 0.04% (Figure S3 and Table 2).

In summary, low concentrations of WD exerted anti-inflammatory activity and maintained the levels of inflammatory markers comparable to the control cells.

### 3.4. Oxidative Stress Was Reduced by Exposure to WD

**Figure 3:**
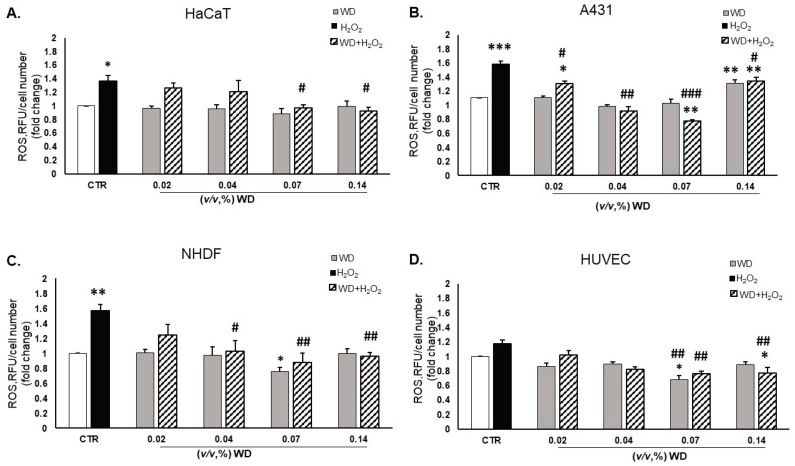
Antioxidant effect of WD on skin cells. HaCaT (**A**), A431 (**B**), NHDF (**C**), and HUVEC (**D**) were treated with WD [0.02–0.14%, (*v*/*v*)], alone (light grey columns) or in combination with H_2_O_2_ [50 µM] (black and white striped columns), and H_2_O_2_ alone (black columns). ROS levels were measured after 1 h by DCFH2-DA. The results are reported as relative fluorescence units (RFUs) corrected for the number of cells. (n = 3) ± SD * *p* < 0.05, ** *p* < 0.01, and *** *p* < 0.001 vs. untreated cells (CTR); # *p* < 0.05, ## *p* < 0.01, and ### *p* < 0.001, compared to H_2_O_2_ [50 µM].

The measurement of ROS levels was performed in a range of brief timelines, namely 30 min, 1 h, and 4 h (Figure 3 and Figure S4). The DCFH2-DA emission was measured in HaCaT, A431, NHDF, and HUVEC after exposure to WD [0.02–0.14%, (*v*/*v*)], alone or in combination with H_2_O_2_ [50 µM] (Figure 3). Exposure to WD alone did not increase oxidative stress. Particularly, WD [0.07%, (*v*/*v*)] showed a slight significant antioxidant effect, per se, after 1 h of exposure in NHDF and HUVEC, compared to untreated cells (Figure 3C,D). 

In this scenario, the results obtained by treating the cells with WD in combination with H_2_O_2_ confirmed the hypothesis of an antioxidant capacity of WD. Indeed, oxidative stress was significantly reduced by WD [0.02–0.14%, (*v*/*v*)] for each timeline considered (Figure 3 and Figure S4), compared to the positive control involving H_2_O_2_. The ability of WD to decrease oxidative stress was strongly visible in the mucosal cell model (A431), in which the decrease in ROS levels was directly correlated with the increase in the WD concentration (Figure 3B). The cytotoxic effect of WD [0.14%, (*v*/*v*)] (seen in [17]) impaired the antioxidant effect of this concentration in A431 models (Figure 3B). The effect of the tested bio-derivative arose better in NHDF, which showed an interesting reduction in ROS levels at the 0.07% (*v*/*v*) concentration, alone or in combination with H_2_O_2_ (Figure 3C). In HUVEC, a protective effect against the oxidative damage induced by H_2_O_2_ was monitored with 0.07–0.14%, (*v*/*v*) concentrations of WD (Figure 3D).

From these data, a general antioxidant effect was demonstrated by WD in different cell types in the cutaneous system.

### 3.5. WD Did Not Affect Cell Migration

**Figure 4:**
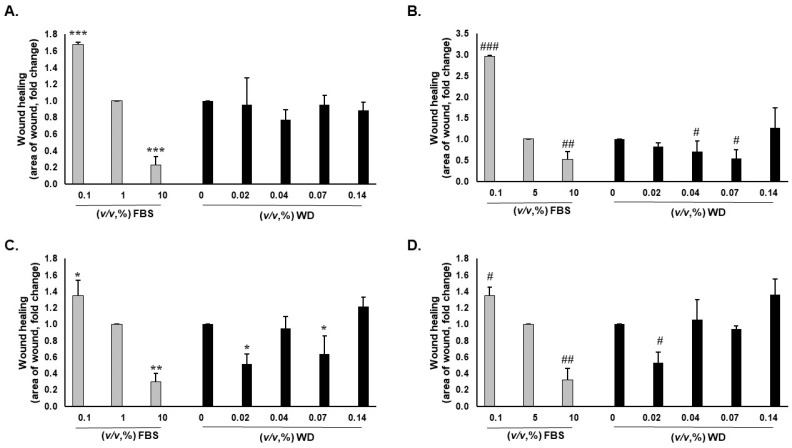
Effect of WD on the mobility of adherent cells. Migration of HaCaT (**A**,**B**) and NHDF (**C**,**D**) was assessed by scratch assay in medium at 1% (**A**,**C**) and 5% FBS (**B**,**D**), after 18 h of exposure to WD [0.02–0.14%, (*v*/*v*)]. Migration was measured as the area of the scratch using FIJI ImageJ software 6.1.1.jar. Data are reported as fold change of the area at 18 h/area at starting time. (n = 3) ± SD * *p* < 0.05, ** *p* < 0.01, and *** *p* < 0.001 vs. cells in medium at 1% FBS (CTR) (**A**,**C**); # *p* < 0.05, ## *p* < 0.01, and ### *p* < 0.001 vs. cells in medium at 5% FBS (CTR) (**B**,**D**).

To investigate the potential role of WD in wound healing, the scratch assay was performed on keratinocytes (HaCaT) and fibroblasts (NHDF), which are critical to the healing process. Cells were responsive to FBS content; thus, a scratch assay was performed with either low (1%) or high (5%) concentrations of serum. Indeed, the scratch area was wider with 0.1% FBS in respect to 10% FBS, which was used as a positive control of migration, inducing the recovery of the cell monolayer (Figure 4). WD was tested in experimental conditions involving 1 and 5% FBS, separately. A not significant modulation of keratinocyte (HaCat) migration was reported, after treatment with WD [0.02–0.14%, (*v*/*v*)], in the low (1%) FBS condition (Figure 4A). An evident healing of the scratch was observed in fibroblasts (NHDF) treated with 0.02 and 0.07% WD in 1% FBS (Figure 4C) and 0.02% with a high concentration of FBS (Figure 4D). 

On the other hand, by increasing the concentration of FBS (5%), cell migration was slightly improved by WD [0.04 and 0.07%, (*v*/*v*)] (Figure 4B). 

Therefore, no clear impairment of cell migration was observed with WD, but a slight reparative effect was evident, even if in a not consistent manner.

## 4. Discussion

This study investigated the effects of *Castanea sativa* wood distillate (WD) on inflammation, oxidative stress, cell viability, endothelial permeability, and wound healing, in various skin cell models. On the whole, the presented work aims to highlight the potential applicability of this natural product in medical human care and the need to deeply investigate the promising support of bio-derivates in new fields of applicability, thus improving the sustainability of the bio-pharma industry. The close relationship between the prolonged exposure to natural products and unexplored impacts on the vascular endothelium, led us to focus on the behavior of endothelial cells treated with diluted WD, monitoring inflammatory markers, permeability, and oxidative stress as pathological and dysfunctional signals.

Building on our previous research that established the safety of diluted WD on keratinocytes (HaCaT), mucosal cells (A431), dermal fibroblasts (NHDF), and endothelial cells (HUVEC) [17], we confirmed that safe concentrations of WD (0.04–0.07%, *v*/*v*) did not induce apoptosis or DNA damage, as indicated by the absence of comet-like structures in an alkaline comet assay.

The high content of organic compounds, particularly tannins (10–13%, *v*/*v*), phenols (3 ± 0.2 g/L), and polyphenols (24 ± 2 g/L), along with the low levels of heavy metals (<1 mg/kg) in WD [20], suggests that it may have potential therapeutic benefits. Therefore, we further explored the anti-inflammatory and antioxidant properties of this bio-derivate. Therefore, safe concentrations of WD were investigated for their anti-inflammatory and antioxidant capacity. In this scenario, the hyperpermeability of endothelium represents a pathological phenomenon caused by high levels of inflammatory stimuli. WD maintained endothelial barrier integrity under inflammatory conditions induced by IL-1 and TNF-α, preventing hyperpermeability. The modulation of proteins involved in tight junctions, VE-cadherin and ZO-1, supported this protective effect. We previously reported that brief or prolonged exposure to WD [0.04–0.07%, (*v*/*v*)] did not induce the expression of COX-2 and mPGES-1 in skin cells [17]. Here, WD reduced the levels of the inflammatory markers, COX-2 and mPGES-1, particularly at a concentration of 0.07%. Moreover, during the inflammatory process, the binding of TNF-α to its receptor induces the expression of adhesion molecules typical of endothelial cells, such as E-selection, ICAM-1, and VCAM-1, needed for the transmigration of leukocytes, recruited at inflammatory sites [1,2]. Physiologically, ICAM-1 and VCAM-1 are slightly expressed by immune and endothelial cells; however, they are over-regulated as a result of inflammatory events [25,26]. Interestingly, WD also attenuated the expression of adhesion molecules, such as VCAM-1 and ICAM-1, suggesting interference with immune–endothelial cell interactions. Overall, these findings indicate a protective effect of WD on endothelial cells.

In the context of oxidative stress, WD exhibited significant antioxidant activity, reducing ROS levels in cells exposed to H_2_O_2_. These data align with the protective effects observed in other natural products [27,28,29]. Indeed, many natural products are already in use to mitigate oxidative and inflammatory damage [13,30,31,32,33,34,35,36,37,38]. Pyroligneous acid extracted from the wood of Fraxinus shows anti-inflammatory action on macrophages exposed to lipopolysaccharides (LPS) [30]. Likewise, extracts from Japanese chestnut exhibit remarkable ROS scavenging capacity [31].

Finally, the potential role of WD in cell migration in in vitro skin cellular models was evaluated to assess the beneficial functional effects of WD on cell health and recovery after damage. WD slightly increased NHDF cell migration, indicating potential benefits for wound healing, especially under inflammatory or infected conditions, due to its anti-inflammatory, antioxidant, and antibacterial properties. These findings are consistent with the protective activity and positive role in treating skin damage of natural extracts or medicinal plants [31,32,33,34,35,36,37,38]. In fact, botanicals are widely used in traditional medicine to treat skin damage, such as cuts, wounds, and burns [33,34,35]. The analysis of many natural extracts and medicinal plants, such as *Curcuma longa*, *Centella asiatica*, *Aloe barbadensis*, *Panax ginseng*, and others, has confirmed their protective activity and their positive role in the treatment of skin damage through their disinfection, antioxidant, and anti-inflammatory action, while also providing a moist atmosphere, which facilitates the development of an appropriate natural healing environment [31,32,33,34,35,36,37,38]. Overall, our data suggest that WD may improve the wound healing process and the recovery of skin homeostasis in conditions of inflammation or infection, through the exerted anti-inflammatory, antioxidant, and antibacterial properties.

## 5. Conclusions

In conclusion, *Castanea sativa* WD, at safe concentrations, offers protective anti-inflammatory and antioxidant benefits, potentially improving wound healing and supporting better skin health and recovery.

The polyhedric action of plant-derived bio-derivates highlights that the potential use of WD in dermatological care needs to be further investigated. Indeed, despite the difficult management and necessity of surveillance in the use of natural extracts or medicinal plants, over the past decades many herbal formulations have been patented for medical care or tissue wellness [38].

The established safety profile, the promising anti-inflammatory and antioxidant activities obtained in skin cells and in the vascular endothelium, corroborate the possibility of finding novel fields of application for WD. The features of this bio-derivative may complement other interventions, namely conventional drugs in skin wellness, by impairing excessive inflammation and oxidative stress. A deeper investigation, standardization, and an optimized formulation of *Castanea sativa* wood distillate could improve its applicability in terms of many physio-pathological conditions, in addition to its consolidated agricultural employment.

## Figures and Tables

**Table 1:** Physio-chemical characterization of wood distillate (BioDea^®^), as indicated by the producer (Bio-Esperia Srl) and reported by Celletti et al., 2023 [20].

Parameter/Chemical Element	Value
TOC (% DW)	58.03
TN (% DW)	1.06
H (% DW)	7.27
S (% DW)	0.07
pH	4
Density (g mL^−1^)	1.05
Flash point (°C)	>60
Total organic compounds (g L^−1^)	33.8
Acidity (mg L^−1^)	1289
Organic acids (mg L^−1^)	32.3
Acetic acid (mg L^−1^)	21.5
Polyphenols (g L^−1^)	24.5
Phenols (g L^−1^)	3
PCBs (mg L^−1^)	<0.2
Hydrocarbons C < 12 (mg L^−1^)	<0.1
Hydrocarbons C10–C40 (mg L^−1^)	<0.1
Elements (mg L^−1^)	
Ca	325.50
Na	103.59
K	23.49
Fe	21.16
P	7.28
Mg	6.79
Zn	3.22
Al	1.96
Mn	0.58
Cu	0.18
Ba	0.06
Cr	0.03
Mo	0.0007

TOC: total organic carbon; TN: total nitrogen; PCBs: polychlorinated biphenyls; Al: aluminum; Ba: barium; C: carbon; Ca: calcium; Cr: chromium; Cu: copper; Fe: iron; K: potassium; Mg: magnesium; Mn: manganese; Mo: molybdenum; Na: sodium; Zn: zinc. The 16 US EPA PAHs is a list of 16 priority polycyclic aromatic hydrocarbons as classified by the United States Environmental Protection Agency. The compounds (Acenaphthene, Acenaphthylene, Anthracene, Benzo[a]anthracene, Benzo[a]pyrene, Benzo[b]fluoranthene, Benzo[g,h,i]perylene, Benzo[k]fluoranthene, Chrysene, Dibenz[a,h]anthracene, Fluoranthene, Fluorene, Indeno[1,2,3-cd]pyrene, Naphthalene, Phenanthrene, Phenanthrene) are <0.05 mg L^−1^.

**Table 2:** Quantification of immunolabelled markers involved in prostanoid cascade, COX-2 and mPGES-1, and leukocyte rolling, VCAM-1 and ICAM-1.

Inflammatory Markers	CTR	MIX	0.04% (*v*/*v*, WD)	0.07% (*v*/*v*, WD)	MIX + 0.04% (*v*/*v*, WD)	MIX + 0.07% (*v*/*v*, WD)
COX-2	0.07 ± 0.006	0.38 ± 0.027 ***↑↑	0.06 ± 0.023 ^###^↓	0.8 ± 0.005 ^###^↓↓	0.10 ± 0.007 ^##^↓	0.07 ± 0.024 ^###^↓↓
mPGES-1	0.33 ± 0.023	0.68 ± 0.039 ***↑↑	0.50 ± 0.010 *^#^↓↑	0.26 ± 0.006 ^###^↓↓	0.34 ± 0.015 ^##^↓	0.37 ± 0.025 ^##^↓
VCAM-1	0.11 ± 0.021	0.52 ± 0.072 ***↑↑	0.20 ± 0.056 ^##^↓	0.13 ± 0.022 ^###^↓↓	0.26 ± 0.014 *^##^↓↑	0.16 ± 0.063 ^###^↓↓
ICAM-1	0.10 ± 0.039	1.08 ± 0.041 ***↑↑	0.27 ± 0.072 *^###^↓↓↑	0.12 ± 0.040 ^###^↓↓	0.10 ± 0.008 ^###^↓↓↑	0.09 ± 0.016 ^###^↓↓

* Immunofluorescence intensity was quantified using FIJI ImageJ software 6.1.1.jar and reported as arbitrary densitometry units (A.D.Us.)/counted nuclei. Data are presented as mean ± SD (n = 3 random images for each condition. * *p* < 0.05, and *** *p* < 0.001 vs. untreated cells (CTR); ^#^ *p* < 0.05, ^##^ *p* < 0.01, and ^###^ *p* < 0.001, compared to MIX. Representative pictures are shown in (Figures S2 and S3). The red arrow represents the increase in inflammatory markers compared to the control conditions (CTR), while the blue arrow represents the decrease compared to the inflammatory conditions (MIX).

## Data Availability

The original contributions presented in the study are included in the article/Supplementary Materials; further inquiries can be directed to the corresponding author/s.

## Supplementary Materials

The following supporting information can be downloaded at: https://www.mdpi.com/article/10.3390/cimb46090556/s1, Figure S1. Permeability assay after prolonged exposure to WD in HUVEC; Figure S2. Immunofluorescence analysis of COX-2 and mPGES-1 in HUVEC; Figure S3. Immunofluorescence analysis of VCAM-Q and ICAM-1 on HUVEC; Figure S4. ROS level measured after 30 min and 4 h by DCFH2-DA.
