# Supplementary material for: Promising Support Coming from Nature: Antioxidant and Anti-Inflammatory Potential of Castanea sativa Wood Distillate on Skin Cells
Source: Curr Issues Mol Biol. 2024 Aug 26;46(9):9386–400. doi: 10.3390/cimb46090556 (PMC11429965; doi:10.3390/cimb46090556)
Supplement: Supplementary file 1 [file cimb-46-00556-s001.zip › cimb-3133038-supplementary.pdf]

Promising Support Coming from Nature: Antioxidant and Anti-Inflammatory Potential of Castanea sativa Wood Distillate on Skin Cells

Arianna Filippelli [1], Valerio Ciccone [1], Stefano Loppi [2,3] and Lucia Morbidelli [1,3,*]

1 Laboratory of Pharmacology of Angiogenesis and Microcirculation, Department Life Sciences, University of Siena, Via A. Moro 2, 53100 Siena, Italy

2 BioAgry Laboratory, Department Life Sciences, University of Siena, Via P.A. Mattioli 4, 53100 Siena, Italy

3 Interuniversity Center for Studies on Bioinspired Agro-Environmental Technology (BAT Center), 80055 Naples, Italy

* Correspondence: morbidelli@unisi.it

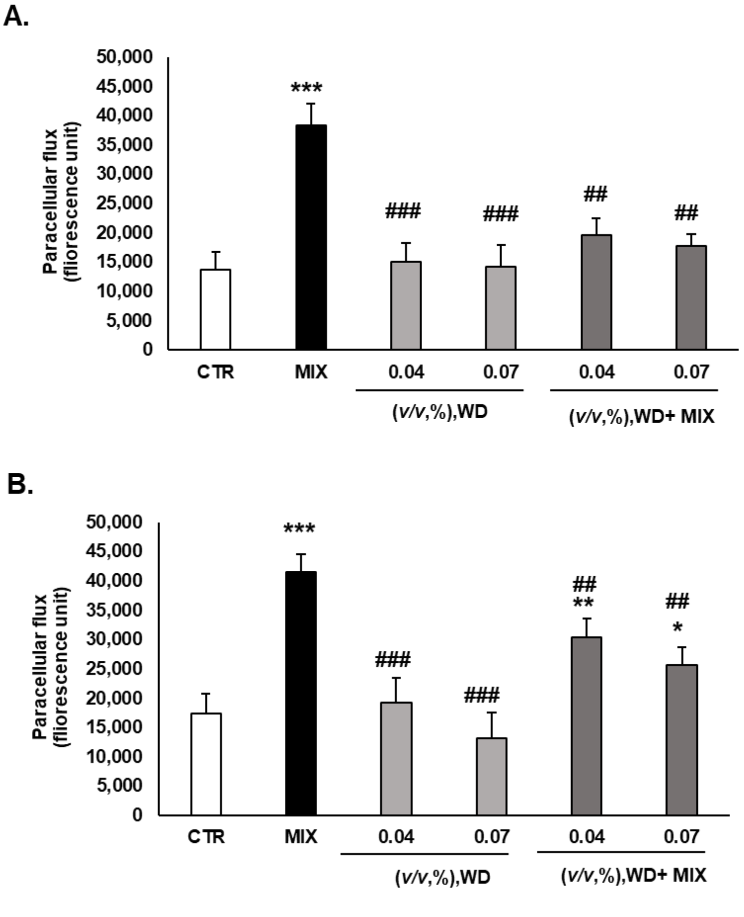

Figure S1: Effect of WD on endothelial paracellular permeability in HUVEC. The permeability assay was performed on HUVEC seeded on transwell inserts. The confluent monolayers were treated with WD [0.04–0.07%, (v/v)] and an inflammatory mix (MIX: IL-1β [100 ng/ml] + TNF-α [10 ng/ml]). At the end of treatment FITC-dextran (10 μM) was added and permeability in HUVEC monolayer was detected as passage of FITC-dextran from upper to lower compartment of the transwell. The extent of permeability was determined by measuring the fluorescence at 485/535 nm, excitation/emission, respectively, after (A)45 minutes and (B)1 hour. Data are reported as fluorescence unit (n=3) ± SD *$p < 0.05$, **$p < 0.01$, and ***$p < 0.001$ vs untreated cells (CTR); # $p < 0.05$, ## $p < 0.01$, and ### $p < 0.001$, compared to MIX.

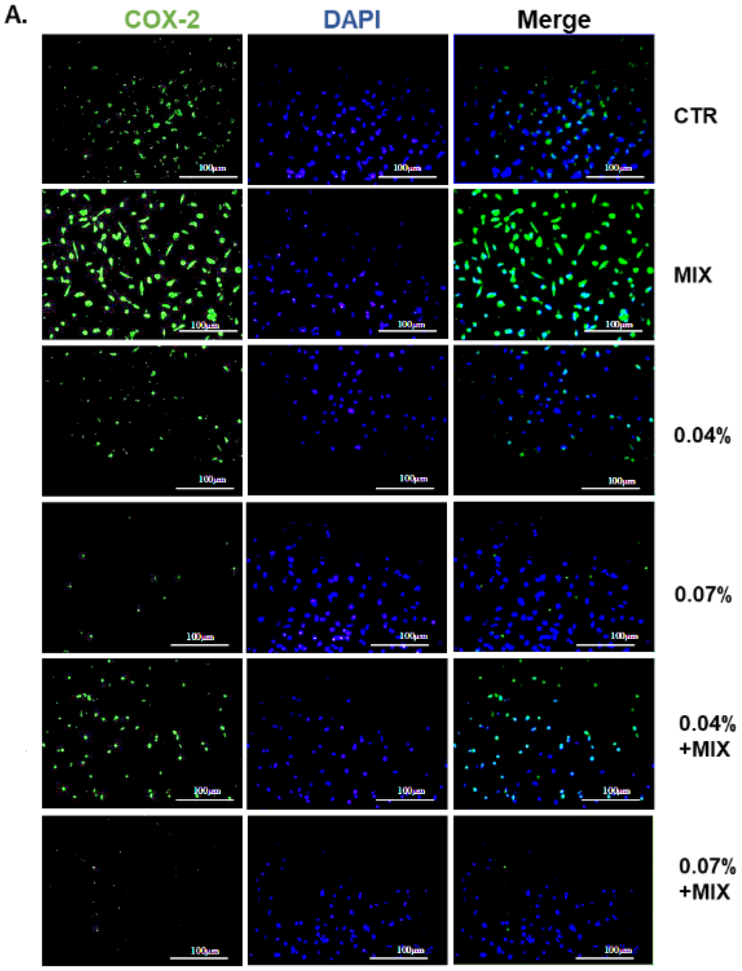
A.
COX-2
DAPI
Merge
CTR
MIX
0.04%
0.07%
0.04% +MIX
0.07% +MIX
100µm

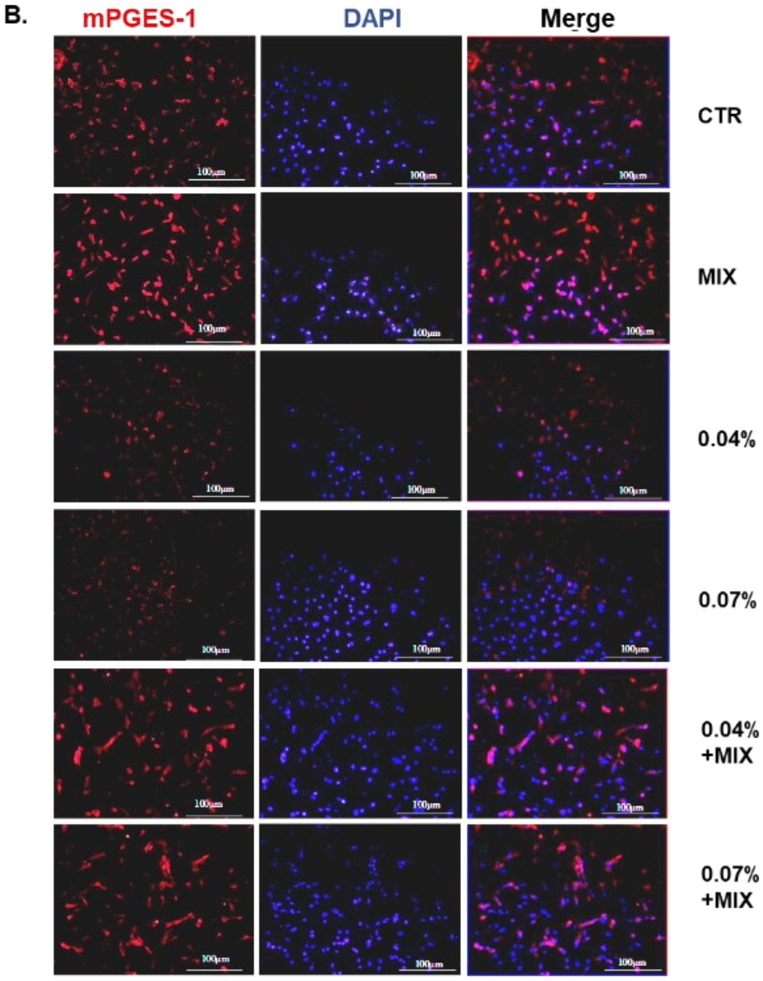

Figure S2. WD negatively modulated inflammatory markers of the prostanoid cascade. COX-2 (A) and (B) mPGES-1 were investigated by immunofluorescence assay in HUVEC. Cells were exposed to WD [0.04-0.07 %, (v/v)] alone or in combination with inflammatory mix (MIX: IL-1β [100 ng/ml] + TNF-α [10 ng/ml]. COX-2 (A) was detected by Alexa Fluor 488, while mPGES-1 (B) was visualized by Alexa Fluor 555. DAPI staining of nuclei in blue. Images were obtained by a Nikon Eclipse TE 300 microscope (magnification 20x). The merge of images was obtained by FIJI ImageJ software. The presented images were reported as representative of n=3 experiments.

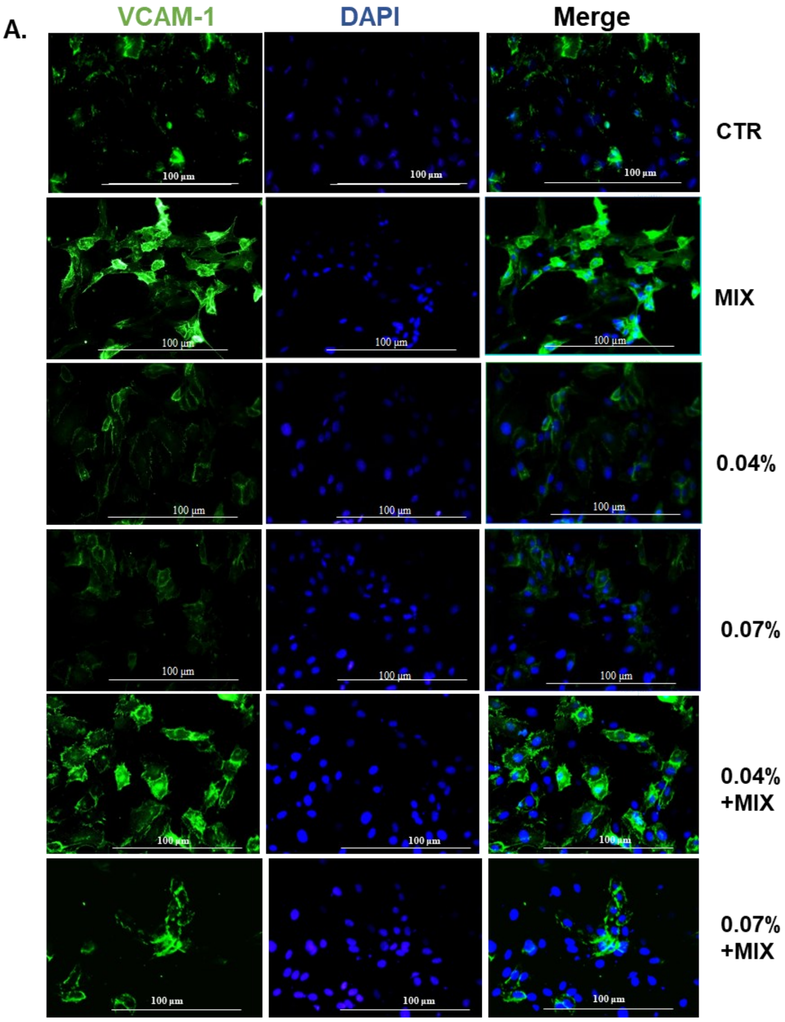
A.
VCAM-1
DAPI
Merge
CTR
MIX
0.04%
0.07%
0.04%
+MIX
0.07%
+MIX
100 µm

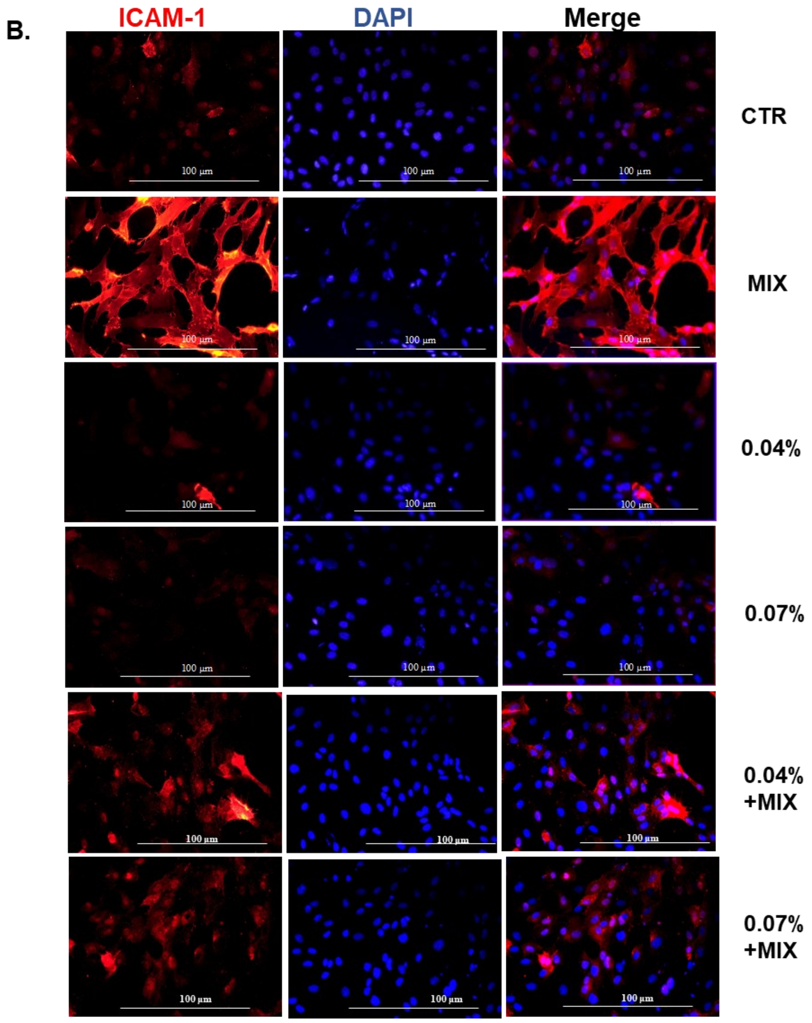

Figure S3. WD did not increase the level of VCAM-1 and ICAM-1 and reduced VCAM-1 and ICAM-1 levels induced by inflammatory stimuli in endothelial cells. Proteins involved in recruitment and rolling of immune cells, VCAM-1 (A) and ICAM-1 (B) were investigated in HUVEC treated with WD [0.04-0.07 %, (v/v)] alone or in combination with inflammatory mix (MIX: IL-1β [100 ng/ml] + TNF-α [10 ng/ml] for 18 hours. VCAM-1 (A) was detected by Alexa Fluor 488, while ICAM-1 (B) was visualized by Alexa Fluor 555. DAPI staining of nuclei in blue. Images were obtained by a Nikon Eclipse TE 300 microscope (magnification 40x). The merge of images was obtained by FIJI ImageJ software 6.1.1.jar. The presented images were reported as representative of n=3 experiments.

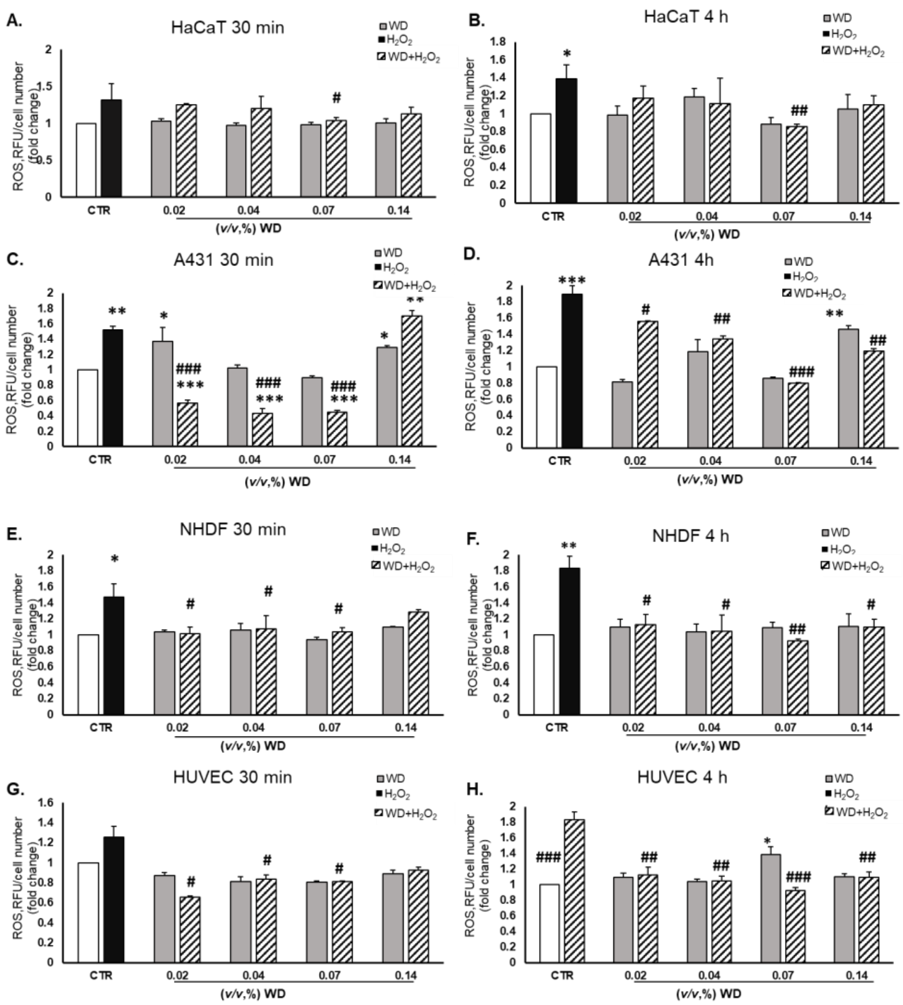

Figure S4. WD exerted an antioxidant effect. HaCaT (A,B), A431 (C,D), NHDF (E,F) and HUVEC (G,H) were treated with WD [0.02-0.14 %, (v/v)] alone (light grey columns) or in combination with H2O2 [50 μM] (black and white striped columns), and H2O2 alone (black columns. ROS levels were measured after 30 minutes (A,C,E,G), 4 hours (B,D,F,H) by DCFH2-DA added in a medium without phenol red (10 μm, 30 minutes). The results are reported as relative fluorescence units (RFU) corrected for the number of cells once fixed, stained and counted. (n=3) ± SD *$p< 0.05$, **$p< 0.01$, and ***$p< 0.001$ vs untreated cells (CTR); # $p < 0.05$, ## $p < 0.01$, and ### $p<0.001$, compared to H_2O_2 [50μM].
